# Higher final speed in 30–15 intermittent fitness tests correlates with soccer's locomotor demands, not heart rate responses in small-sided soccer games

**DOI:** 10.1038/s41598-024-61468-7

**Published:** 2024-05-14

**Authors:** YanXiu Quan, YongXing Zhao, XiaoShuang Wang, Qi Xu

**Affiliations:** 1 College of Physical Education, China West Normal University, Nanchong, 637009 Sichuan China; 2 College of Physical Education, Chizhou University, Chizhou, 247000 Anhui China; 3 Gdansk University of Physical Education and Sport, 80-336 Gdańsk, Poland

**Keywords:** Football, Athletic performance, Training load, Conditioned games, Drill-based games, Health care, Health occupations

## Abstract

This study aimed to achieve two objectives: firstly, to analyze the relationships between aerobic fitness, as represented by the VIFT, and the heart rate and locomotor responses of youth male soccer players across various teams; and secondly, to compare players with lower and higher VIFT in terms of performance outcomes extracted during small-sided games (SSGs). A total of twenty-six youth male soccer players, aged 16.5 ± 0.32 years, with 3.4 ± 1.1 years of experience, voluntarily participated in the study. These players belonged to two regional-level tier 2 teams (trained/developmental). In the initial week of observation, the 30–15 Intermittent Fitness Test was implemented to measure the final velocity (VIFT) achieved by the players. Subsequently, the 5v5 format of play was conducted twice a week over two consecutive weeks, during which heart rate responses and locomotor demands were measured. The Pearson product-moment correlation test revealed a significant correlation between VIFT and the total distance covered during the 5v5 format (r = 0.471 [95% CI: 0.093; 0.721], *p* = 0.015). Conversely, small and non-significant correlations were identified between VIFT and mean heart rate (r = 0.280 [95% CI: − 0.126; 0.598]; *p* = 0.166), VIFT and peak heart rate (r = 0.237 [95% CI: − 0.170; 0.569]; *p* = 0.243), as well as VIFT and high-speed running (r = 0.254 [95% CI: − 0.153; 0.580]; *p* = 0.211). Players with higher VIFT demonstrated a significantly greater total distance, with a large effect size (+ 6.64%; *p* = 0.015; d = 1.033), compared to those with lower VIFT. Our findings suggest that improved performance in VIFT may lead to covering more distance in 5v5 matches. However, the lack of significant associations between VIFT and heart rate levels during SSGs suggests that they are not strongly correlated, possibly because VIFT is more closely linked to locomotor profile. As a practical implication, coaches may consider organizing players during SSGs based on their VIFT if the goal is to standardize locomotor demands.

## Introduction

Small-sided games (SSGs) represent well-established and popular training drills that simplify the complexities of real game formats with the aim of emphasizing specific objectives, such as tactical, technical, physical, and physiological aspects^1^. These games ensure the maintenance of the fundamental properties and dynamics of the game, such as one team opposing another^2^. While preserving these core properties, SSGs provide an opportunity to introduce acute stimuli, as players adapt their behavior to the dynamic scenarios and context, thereby naturally influencing locomotor demands^3,4^. However, given that environmental, task, and individual constraints shape performance outcomes, it is anticipated that inter-player variability in performance during SSGs may be influenced by individual factors, including tactical capacity^5^, technical skill proficiency^6^, and physical fitness status^7^.

SSGs can be designed and implemented in various ways by manipulating a range of task constraints, including numerical relationships (i.e., format of play), pitch configuration, specific rules (e.g., limitations on ball touches, time constraints to achieve a specific goal), or task objectives (e.g., scoring in small goals, achieving a set number of consecutive passes)^8,9^. These task constraints act synergistically to determine acute player responses^10,11^. For instance, when comparing smaller formats played on larger fields to larger formats in smaller spaces, a notable increase in metabolic costs, evidenced by higher energy expenditure, is observed^12^. This is evident through a significant rise in the amount of time spent in heart rate zones above 85% of maximal heart rate^13^. Additionally, there is a significant increase in the total distance covered and mechanical work undertaken during these smaller formats, exemplifying the impact of task constraints on player performance^14^.

While acute responses to SSGs can be influenced by task constraints and the design of the games, it is also reasonable to expect that individual differences among players can significantly impact their overall performance in SSGs^1,15^. This is particularly relevant in certain SSG formats, such as 5v5. Perhaps this format is frequently utilized in training sessions due to the depth of tactical behaviors that can be explored^16^. Additionally, it elicits intense physiological and locomotor responses, making it imperative to pay special attention to the variables that can impact player performance within this format^17^. One of these factors is physical fitness, which plays a role in how players meet the challenges posed by SSGs^18^. For instance, a study conducted with male professional players demonstrated substantial and meaningful correlations between the final velocity achieved in the 30–15 Intermittent Fitness test (VIFT) and various locomotor parameters, including total distance, high-intensity running, and mechanical work performed in a 3v3 format of play^7^. Another descriptive study involving professional male soccer players revealed potentially small to very large relationships between performance in the Yo-Yo Intermittent Recovery test and running performance during a 5v5 format of play^19^. Additionally, a study conducted with youth players unveiled significant correlations between anaerobic speed reserve and VIFT with the total distance covered in a 5v5 format of play^20^. These findings emphasize the intricate interplay between individual physical fitness levels and on-field locomotor performance in the context of SSGs.

However, the interaction between physical fitness and heart rate responses during SSGs remains relatively unexplored. In a study conducted by Younesi and colleagues^7^, meaningful correlations between the 30–15 Intermittent Fitness test (VIFT) and heart rate responses were not identified. Conversely, hemoglobin levels demonstrated significant and moderate to large correlations with heart rate-derived variables^7^. Despite heart rate responses being a commonly studied variable in SSGs, consistently stable across different repetitions and maintaining consistently high-intensity levels across various format sizes, ranging from 1v1 to 5v5^21^, little is known about the relationship between aerobic physical fitness and acute responses in SSGs.

Understanding how individual physical fitness, particularly aerobic fitness, can influence player responses to SSGs is crucial for grouping players effectively and considering individuality as a constraint that modulates overall performance in these games. Adding a better understanding of how aerobic fitness relates to heart rate responses and locomotor demands during SSGs can ultimately guide coaches in managing players during these games and adopting strategies for a more individualized training approach, especially for those who may not express higher demands in these contexts.

It is noteworthy that all existing studies examining relationships between physical fitness and acute responses during SSGs have been limited to a single team, thus being constrained by the specificities of the sampling. For better generalization, beyond having a larger sample size, it is essential to gather data from different teams to determine whether these relationships are context-dependent. For instance, within a team, the heterogeneity of crucial physical variables like VIFT can influence the outcomes of an acute response, potentially shaping the overall distances covered by players in a specific SSG or their acute physiological responses such as heart rate^7,22^. Other factors, both physical and technical/tactical, can also influence individual responses to the same SSG format^23^.

Recognizing the significance of addressing these gaps in current knowledge, this study aims to analyze the relationships between aerobic fitness, represented by the VIFT, and the heart rate and locomotor responses of youth male soccer players belonging to various teams. Moreover, the study aims to compare players with lower and higher VIFT regarding their heart rate responses and the total distance covered during SSGs. Supported on previous findings^7,19^, it is anticipated that VIFT will be meaningfully associated with total distance and distances covered at high-intensity levels. Furthermore, it is expected to observe relationships between VIFT and maximal and mean heart rates exhibited by the players. Additionally, we hypothesize that players with higher VIFT will exhibit significantly higher average heart rate responses during SSGs and cover significantly greater distances compared to those with smaller VIFT.

## Methods

### Participants

The a priori sample size was determined by considering the minimum effect size identified in a reference study that correlated VIFT and locomotor demands (effect size = 0.56), with a set significance level of 0.05 and a power of 0.85. Utilizing G*power (version 3.1.9.6.) for the correlation test, the recommended sample size was established at 18 players. The eligibility criteria were defined based on the following points: (i) participation in all observed training sessions, without missing reports on the 5v5 format and evaluation of VIFT; (ii) absence of injuries during the last month of the observed period and no occurrence of illness throughout the observation period; (iii) exclusive inclusion of outfield players, excluding goalkeepers. A convenience sampling strategy was employed, and the recruitment process commenced by reaching out to under-17 male youth soccer teams participating in the region's competition. Invitations were extended via email, and clubs that responded positively were subsequently invited to learn about the experiment. Upon the agreement of two clubs, the main researcher personally approached the players within these clubs. The purpose was to explain the experimental procedures and outline the ethical standards guiding the research.

Despite thirty participants initially declaring their availability to participate in the experiment, four of them failed to complete one or more sessions during the observation period and were consequently excluded from the analysis. Twenty-six youth male soccer players (aged 16.5 ± 0.32 years, with 3.4 ± 1.1 years of experience; 1.74 ± 3.1 cm; 62.4 ± 2.7 kg) from two regional-level tier 2 teams (trained/developmental) voluntarily took part in the study. On average, participants engaged in three training sessions per week, along with participating in an official match during the weekends. The study adhered to the ethical standards of the Declaration of Helsinki. It received approval from the Chengdu Institute of Physical Education ethical committee (2023#104), and the participants, along with their parents, were informed about the study. They willingly signed a free and informed consent form.

### Study design and experimental approach

This study employed an observational study design. The 5v5 format of play was performed twice a week over two consecutive weeks. The observations took place two months after the beginning of the season, specifically during the early in-season period. In the week preceding the observation of the 5v5 games, the players underwent an aerobic fitness assessment. Specifically, on Tuesday, following a two-day interval from the last competitive match, they were subjected to the 30–15 Intermittent Fitness Test. The final velocity attained in the test (VIFT) was correlated with both heart rate and locomotor demands recorded during the 5v5 formats played throughout the observation period.

### The 30–15 Intermittent Fitness Test

The original 30–15 Intermittent Fitness Test^24^ was employed to evaluate the aerobic fitness of the participants. The test was chosen due to its capacity to assess the locomotor performance of soccer players reliably^25,26^. This choice was influenced by its demonstrated high reliability levels, as indicated in a previous study^27^ which revealed an intra-class correlation test of 0.80 for between-session reliability and a coefficient of variation of 2.5%. The test took place 48 h after the most recent match, with the assessment occurring at 5 pm under temperature conditions of 21.3 ± 0.5°C and relative humidity of 32.1 ± 1.2% on a synthetic turf. Following a 5-min self-paced jogging warm-up, all participants were grouped to undergo the test. This test involves 30-s intervals of intense running followed by 15 s of active recovery. The pattern repeats, with the running speed gradually increasing in successive stages. The initial speed is set at 8 km/h. Throughout the test stages (30 s each), the running speed increases by 0.5 km/h until the participant can no longer keep up with the pace signaled by an audio cue. The final velocity (stage) completed by the player (VIFT) was recorded as the primary outcome of aerobic performance for subsequent data analysis.

### Small-sided games

Following a standardized warm-up protocol based on the FIFA 11 + guidelines^28^, players engaged in two sets of 5-min 5v5 SSGs with small goals, separated by 3 min of rest. These game sessions occurred on both Tuesday (2 days after the last match) and Thursday (4 days after the last match) of each week, with a one-day rest period in between. The training sessions commenced at 5 pm, and the games were executed on a synthetic turf, under dry weather conditions, with a temperature of 19.3 ± 1.1°C and a relative humidity of 34.2 ± 2.3°C. The SSGs were the unique focal point of the training analyzed and preceded the remaining regular training practices planned by the coach which were not analyzed.

The 5v5 SSG format was implemented on a pitch measuring 50 × 30 m, featuring small goals (2 × 1 m) positioned at the center of the end lines. The dimensions were chosen based on previous recommendations for this format of play^29^. Match rules excluded the offside rule, and ball repositioning was executed using the foot. To expedite ball repositioning when the ball went out of limits, two balls were strategically placed along the pitch lines. No verbal encouragement was provided. The players engaged in two five-minute repetitions with a 3-min rest interval between each. This identical regimen was consistently applied in every analyzed training session.

The coach assigned players to each team, ensuring a balanced distribution based on their playing positions and skill proficiency. Teams and opponents remained constant across different repetitions and sessions to minimize contextual variability. Before the games commenced, participants were encouraged to adopt their preferred strategies but were motivated to exert maximal effort throughout the matches.

### Player’s monitoring

The players were systematically monitored in all matches using a microelectromechanical system that integrates a Global Navigation Satellite System and a heart rate sensor. The unit system (10 Hz, Polar Team Pro, Polar, Finland) was tightly positioned on the chest of each participant using a dedicated band. This system, previously described for its accuracy and precision^30–32^, was employed to measure both locomotor demands and heart rate measures. For each of the 5v5 repetitions, the following outcomes were recorded: average heart rate (avHR; beats per minute), peak heart rate (HRpeak; beats per minute), total distance covered (meters), distance covered between 15.00 and 18.99 km/h (high-intensity running; meters), and distance covered > 19 km/h (high-speed running; meters). To standardize the outcomes, the locomotor demands were segmented by minute. During the analysis period, each player was monitored in eight sets of 5-min 5v5 SSGs, resulting from two sets per session multiplied by two sessions performed over the course of two weeks. The averages of all repetitions and sessions were used for subsequent data analysis.

### Statistical procedures

Data normality and homogeneity were assessed using Shapiro–Wilk and Levene’s tests, respectively, with p-values > 0.05, allowing for the application of further parametric tests. The within-players variability of heart rate and locomotor responses across different sessions was expressed as the average coefficient of variation (%). Descriptive statistics, including mean and standard deviation, were presented.

To establish the magnitude of the relationship between VIFT and heart rate and locomotor variables obtained during the 5v5 format, the Pearson-product correlation test was employed. The correlation magnitudes were interpreted based on Cohen’s criteria^33^: < 0.1, no effect; 0.1 ≤ r < 0.3, small; 0.3 ≤ r < 0.5, medium; r ≥ 0.5, large effect.

To compare players with higher and lower VIFT values, the players were categorized based on the median of the group, which was 15 km/h. Those below 15 km/h were classified as having a lower VIFT, while those above were classified as having a higher VIFT. The independent t-test was conducted to assess differences between players with lower VIFT (< 15.0 km/h) and higher (≥ 15.0 km/h) concerning outcomes obtained during the 5v5 format. Cohen's d was also calculated as a measure of effect size in pairwise comparisons. The magnitudes of effect size were categorized following Cohen's recommendations^33^: small (d = 0.2), medium (d = 0.5), and large (d ≥ 0.8). All statistical analyses were conducted using the SPSS statistical software (version 29.0.0.0, IBM, USA) with a significance level set at *p* < 0.05.

#### Ethical approval

The participants willingly provided written consent by signing a consent form. The study was conducted in adherence to the ethics committee of the Chengdu Institute of Physical Education (2023#104) and the principles outlined in the Declaration of Helsinki.

## Results

Table 1 provides details on means, standard deviations, and data resulting from inferential statistics comparing players with lower and higher VIFT. Players with higher VIFT exhibited a significantly greater total distance, with a large effect size (+ 6.64%; p = 0.015; d = 1.033), compared to those with lower VIFT. No other statistically significant differences were observed.

**Table 1:** Descriptive statistics (mean ± standard deviation) for the 5v5 match outcomes, comparing players with lower and higher final velocity at 30–15 Intermittent Fitness Test.

	Lower VIFT (mean ± SD)	Higher VIFT (mean ± SD)	Mean difference (%)	*p*-value	Effect size (d)
HR average (bpm)	165.8 ± 6.2	169.5 ± 5.1	2.24 ± 9.43	*p* = 0.102	d = 0.667 [95%CI: –0.131;1.451]
HR peak (bpm)	184.3 ± 4.4	186.2 ± 3.7	1.03 ± 9.43	*p* = 0.258	d = 0.454 [95%CI: –0.330;1.229]
Total distance (m/min)	99.5 ± 6.1	106.1 ± 6.7	6.64 ± 9.43	*p* = 0.015*	d = 1.033 [95%CI: 0.202;1.846]
High-intensity running (m/min)	7.1 ± 1.8	8.2 ± 1.9	15.49 ± 9.43	*p* = 0.152	d = 0.580 [95%CI: –0.211;1.361]
High-speed running (m/min)	2.3 ± 1.3	2.8 ± 1.4	21.74 ± 9.43	*p* = 0.377	d = 0.342 [95%CI: –0.412;1.089]

VIFT, final velocity at 30–15 Intermittent Fitness Test; HR, heart rate; SD, standard deviation; bpm, beats per minute; m, meters; min: minute; d, cohen’s d value.

*significantly different for a *p* < 0.05.

**Figure 1:**
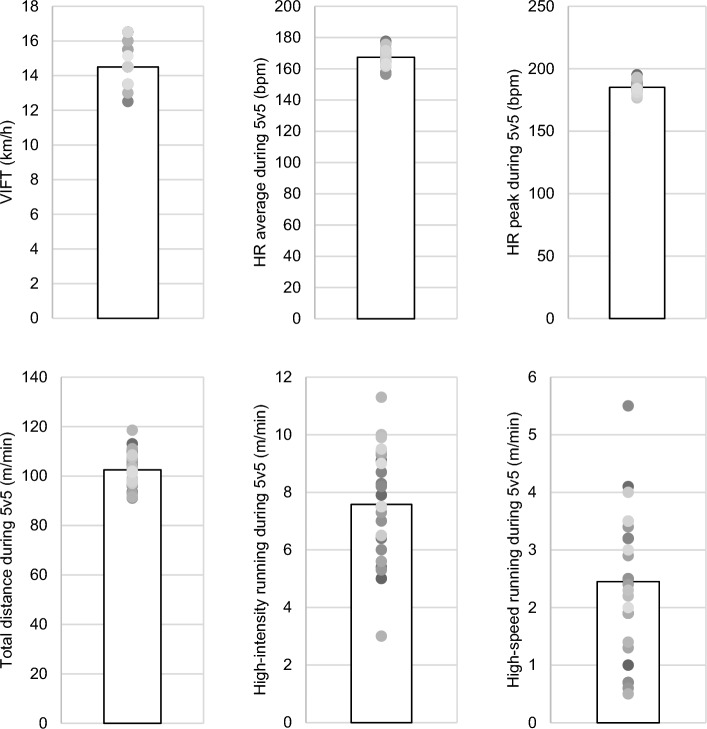
Descriptive statistics illustrating individual players' responses in terms of heart rate (HR) and locomotor responses during the 5v5 gameplay format and the 30–15 Intermittent Fitness Test (VIFT).

Figure 1 displays the descriptive statistics for the player's 30–15 Intermittent Fitness Test (VIFT) and their responses to the demands of the 5v5 match, illustrating various observed outcomes. The average within-player variability (expressed as a percentage of the coefficient of variation for all eight repetitions of the format conducted across two sets, two sessions, and two weeks) for different outcomes during the 5v5 format were as follows: HRaverage (7.5 ± 2.6%), HRpeak (5.7 ± 2.5%), total distance (16.5 ± 4.0%), high-intensity running (51.8 ± 23.5%), and high-speed running (86.0 ± 36.0%).

**Figure 2:**
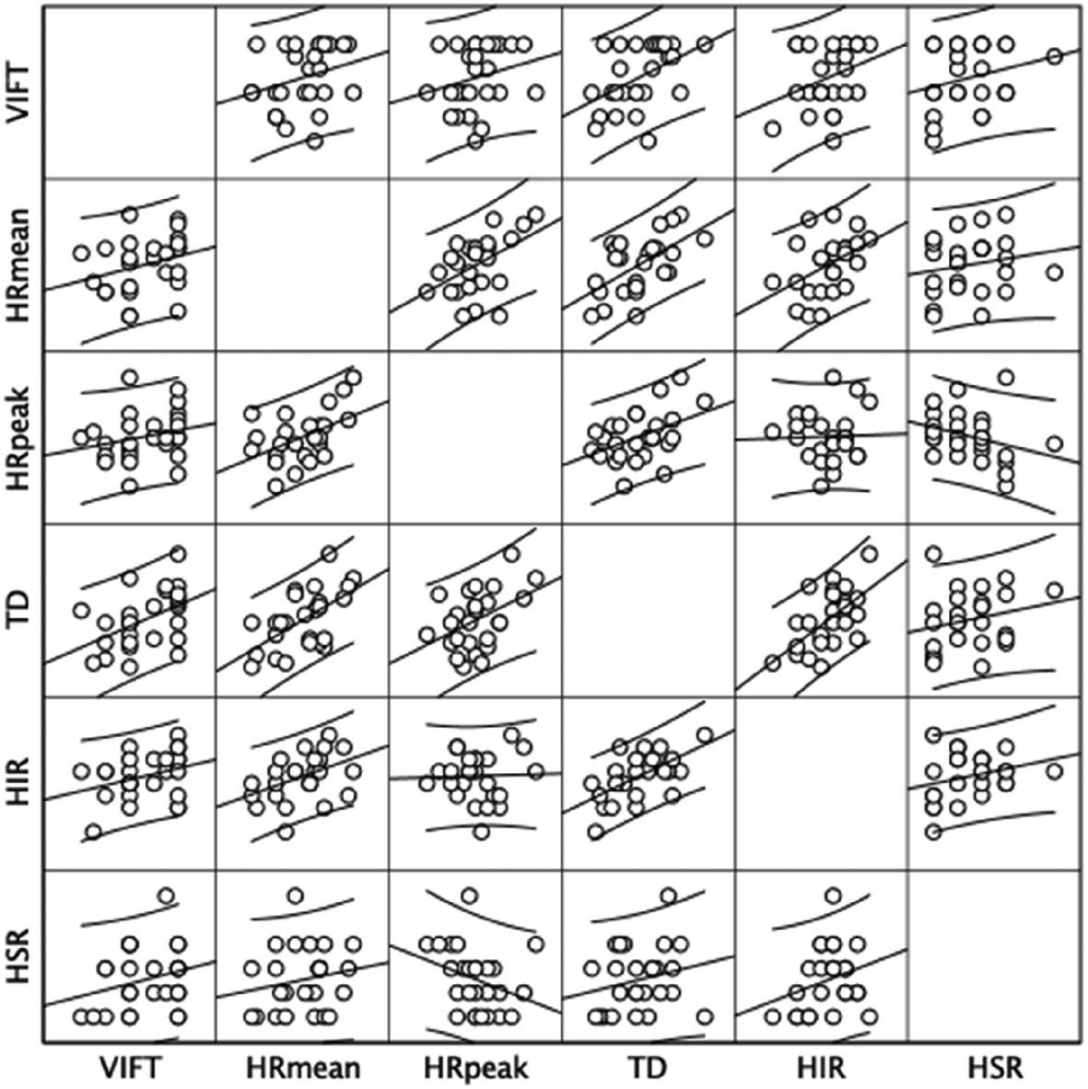
Scatterplot illustrating the relationships between outcomes. VIFT: final velocity at 30–15 Intermittent Fitness Test; HR: heart rate; TD: total distance; HIR: high-intensity running; HSR: high-speed running.

The Pearson product-moment correlation test indicated a significant correlation between the 30–15 Intermittent Fitness Test (VIFT) and the total distance covered during the 5v5 format (r = 0.471 [95% CI: 0.093; 0.721], representing a medium magnitude of correlation; *p* = 0.015). Furthermore, a medium magnitude of correlation was observed between VIFT and high-intensity running, although it did not reach statistical significance (r = 0.317 [95% CI: − 0.086; 0.624], medium magnitude of correlation; *p* = 0.114). Conversely, small and non-significant correlations were identified between VIFT and mean heart rate (r = 0.280 [95% CI: − 0.126; 0.598], small magnitude of correlation; *p* = 0.166), VIFT and peak heart rate (r = 0.237 [95% CI: − 0.170; 0.569], small magnitude of correlation; *p* = 0.243), as well as VIFT and high-speed running (r = 0.254 [95% CI: − 0.153; 0.580], small magnitude of correlation; *p* = 0.211). Figure 2 depicts the relationships between outcomes through scatterplots.

## Discussion

Our current research only partially confirms our hypothesis that VIFT is significantly correlated with the total distance covered by players during SSGs, while it does not confirm our hypothesis regarding the association between VIFT and heart rate responses during SSGs. Furthermore, our hypothesis that players with greater VIFT will cover significantly greater total distance and exhibit higher heart rate responses during SSGs is only partially confirmed, as it was observed in total distance but not in heart rate responses. The present research has revealed a positive association between higher aerobic fitness and greater distance covered in the 5v5 format. Moreover, the study identified a significant and moderately strong correlation between VIFT, and the distance covered during 5v5 formats. Despite observing other medium-magnitude correlations between VIFT and high-intensity running, these were not statistically significant. Measures such as heart rate responses and high-speed running exhibited small and no significant correlations with VIFT. Furthermore, when comparing players with smaller and higher VIFT concerning the mentioned outcomes, no significant variations were found.

The final velocity achieved in the 30–15 Intermittent Fitness Test (VIFT) serves as a metric capable of characterizing players' locomotor performance^25^. This measure depends not only on aerobic capacity^34^ but also on additional factors, such as change-of-direction ability and lower limb strength, which appear to influence better performance in VIFT^35^. As a marker of locomotor performance, it is postulated that achieving higher levels in VIFT may contribute to sustaining elevated locomotor efforts during match situations^7^. Consistent with earlier studies that unveiled noteworthy correlations between aerobic fitness markers such as VIFT^7,20^ or distance covered in the Yo-Yo Intermittent Recovery test^19^, our present research affirms a significant and moderately sized correlation between VIFT and the total distance covered by youth male soccer players in the 5v5 format of play.

A higher VIFT suggests superior aerobic fitness and anaerobic power, enabling the player to sustain and recover from intense efforts more effectively^36^. In SSGs, where players engage in frequent bursts of intense actions (e.g., accelerations, decelerations, intensity running), those with a superior aerobic capacity can maintain a higher work rate throughout the game^37^. The increased endurance resulting from higher VIFT levels enables soccer players to cover more extensive distances, thereby enhancing their overall performance in matches. This correlation is substantiated by research^38^ demonstrating a robust association between heart rate recovery following a standardized 4 vs. 4 SSGs and endurance performance in a controlled laboratory setting. The study^38^ specifically investigated semi-professional soccer players, highlighting the practical relevance of superior aerobic capacity in small-sided game scenarios and reinforcing the critical role of aerobic fitness in optimizing on-field capabilities.

While not exhibiting a statistically significant difference between individuals with higher and lower VIFT, the presence of a medium effective size in correlation with high-intensity running suggests a potential explanation. It is possible that an elevated VIFT level may contribute to enhanced performance during 5v5 games by facilitating sustained efforts throughout the matches since previous studies revealed that better VIFT explains the ability to perform repeated sprinting efforts^39^. This could be attributed to the improved ability to recover swiftly and endure greater intra-game exertions^36^, implying that the relationship between VIFT and performance extends beyond mere statistical significance and may be practically relevant in the context of repeated efforts within the dynamic environment of 5v5 soccer games.

In alignment with prior research that failed to identify significant relationships between VIFT and heart rate responses during SSGs^7,20^, our study similarly uncovered no noteworthy correlations between VIFT and heart rate responses in 5v5 games. Furthermore, our findings indicated a lack of significant differences in heart rate responses during SSGs between individuals with lower and higher VIFT levels. These consistent results across different game formats suggest that, at least in our study population, VIFT may not be a prominent factor influencing heart rate responses during 5v5 games. The lack of a significant relationship between VIFT and heart rate responses during SSGs may be attributed to the multidimensional nature of physiological responses during SSGs. While VIFT estimates an individual's capacity for high-intensity intermittent exercise, heart rate responses in SSGs are influenced by a combination of factors, including team dynamics and interactions with situational factors^40^. As reported in a systematic review concerning physical and tactical factors in football utilizing positional data^41^, medium-sided games such as 5v5 can meaningfully differ from smaller and larger ones in terms of player distribution across the field. They also facilitate a proactive dynamic by encouraging individual actions and promoting variability in player participation within the match. Consequently, the specificity of 5v5 games can influence the relationship between VIFT and physiological impact. Additionally, incorporating tactical analysis based on positional data to elucidate player behavior and responses can be particularly interesting. The absence of a direct correlation may imply that VIFT alone does not encapsulate the complexities of cardiovascular responses during the SSGs. Additionally, variations among individuals in fitness profiles and playing styles among participants could contribute to the lack of a clear association.

The present research is not without its limitations. One notable constraint is the absence of the integration of factors such as tactical behavior and technical demands, which prevents a comprehensive encapsulation of the dynamic processes leading to locomotor and physiological outcomes. It is essential to recognize that the complexity of SSGs extends beyond singular aspects like physical fitness. Future research endeavors should adopt a more holistic approach by incorporating multiple outcomes and establishing relationships with a broader range of variables. This strategy aims to unravel the complex interplay among physical fitness levels, tactical, and technical proficiency, offering a more nuanced understanding of how these elements collectively contribute to a player's commitment and performance in SSGs. For instance, future research should explore the influence of aerobic, anaerobic, and neuromuscular capacities on various formats of SSGs. Additionally, it should research how the magnitude of correlation varies across different competitive levels. Moreover, researchers should consider information regarding tactical expertise and technical proficiency. Furthermore, monitoring responses should incorporate not only locomotor variables such as accelerations, decelerations, high-speed running, and sprinting but also tactical and technical responses during matches.

Notwithstanding its limitations, this study transcends the typical constraint associated with SSGs research by being conducted across two distinct teams. This approach not only ensures a more extensive and diverse sample size but also captures different contextual factors. The results, albeit somewhat confirmatory of previous studies^7,19,20^, suggest a correlation between enhanced physical fitness and increased distance covered in these games. Coaches should take note of this, particularly in assessing whether individuals with lower VIFT are meeting their performance standards during SSGs. Additionally, there is worth in considering standardization in team organization, aligning player intensities based on their fitness levels, thereby optimizing overall team performance in SSGs. Eventually, players should be grouped into SSG formats based on their physical abilities, while incorporating task constraints that adjust the level of participation by aligning with their VIFT levels.

## Conclusions

Our research shows that higher aerobic fitness, measured by VIFT, is linked to covering more distance in 5v5 soccer. VIFT correlates moderately with distance covered, suggesting it is well related with on-field performance. However, correlations between VIFT and high-intensity running are less clear. Other measures like heart rate and high-speed running show weak connections with VIFT. Comparing players with different VIFT levels did not reveal significant differences in performance measures, indicating a complex relationship between aerobic fitness and performance in 5v5 soccer. Our study suggests that grouping players based on VIFT could be particularly interesting when coaches seek to standardize the total distance covered.

## Data Availability

The datasets generated during and/or analysed during the current study are available from the corresponding author on reasonable request.
